# Composing adult lives with a ventilator at the intersection of developmental and neoliberal discourses of time

**DOI:** 10.1177/13634593241226646

**Published:** 2024-01-17

**Authors:** Elizabeth J Straus, Helen Brown, A. Fuchsia Howard, Gail Teachman

**Affiliations:** University of British Columbia, Canada; University of Guelph, Canada; University of British Columbia, Canada; Western University, Canada

**Keywords:** aging and lifecourse, chronic illness and disability, discourse analysis, health, narrative analysis, technology in healthcare

## Abstract

This paper explores temporalities and experiences of time drawn from an analysis of interview data from a critical narrative inquiry of the experiences of young adults living with home mechanical ventilation (HMV). The analysis centers the ideological effects of dominant discourses that shape understandings of time in the Euro-Western world and the ways in which young adults’ stories prompt a rethinking of time in health research and praxis. Data generation involved interviews and photo-elicitation with five young adults (ages 18–40). A critical narrative analysis of participants’ stories surfaced the influence of ableist, developmentalist, and neoliberal discourses of time and the creative resistance that points to the potential of crip orientations to time in opening up possibilities for living. Implications for practice and research are offered.

## Introduction

It is widely acknowledged that medical technologies are extending the lives of young adults living with neuromuscular conditions, spinal cord injuries, and other complex chronic conditions with the use of home mechanical ventilation (HMV) technology—long-term use of ventilation via tracheostomy, non-invasive bilevel positive airway pressure (BiPAP), or mouthpiece—increasing substantially over the last two decades (Povitz et al., 2018; Rose et al., 2015). In this context of extending life with HMV, goals of care often focus on enabling those living with HMV to live as well as possible. Yet, there is a dearth of research focused on theorizing living well with HMV (Israelsson-Skogsberg et al., 2018; Noyes, 2006).

While little research has explored experiences of living well with young adults (ages 18–40 years) living with HMV, researchers have increasingly sought to understand experiences with and meanings of HMV technology. These studies have emphasized how HMV has the potential to increase (through decreasing symptoms associated with respiratory insufficiency) or reduce (e.g. through ventilator technology limitations, access barriers, changes in communication, and feelings of loss of control and autonomy) educational, social, and vocational opportunities (Briscoe and Woodgate, 2010; Israelsson-Skogsberg et al., 2018; Tsay et al., 2013). For many the ventilator becomes part of them, embodied in how they live in the world (Ballangrud et al., 2009; Dreyer et al., 2010). While it is clear that HMV use can shape daily life in myriad ways, few studies attend to the connections between these experiences and living well as young adults.

This paper presents an analysis of interview data from a critical narrative inquiry of the experiences of young adults living with HMV. We employed theoretical principles of critical discourse analysis, specifically considering how power, ideology, and discourse interrelate in analyzing the narrative and story-based data. Across all interviews, participants shared experiences of time and temporalities when living with HMV. The analysis presented here focused on these key themes; that is, how neoliberal and developmentalist discourses are shaping constructions and experience of time and temporality within participants’ stories. We discuss the implications of this analysis and findings for prompting a rethinking of time and living well in health research and praxis.

### Orienting to time in Euro-Western societies

In research and praxis, understandings of and orientations to time are often left unquestioned. In the context of research with disabled people, including those living with HMV, we contend that time must be considered beyond seconds, minutes, hours, days, and years (clock time). How we understand time is shaped, in part, by dominant discourses, such as ableism, developmentalism, and neoliberalism, which can *mask* and *normalize* taken-for-granted assumptions about time and what should be done in time, which can create and sustain negative impacts, such as exclusion, and constrain opportunities for those whose temporalities differ from what is considered *normal*. Disability scholar Kumari-Campbell (2009) describes ableism as “a network of beliefs, processes and practices that produces a particular kind of self and body (the corporeal standard) that is projected as the perfect, species-typical and therefore essential and fully human” (p. 44). In upholding this privileging of the walking, independently breathing, normative bodymind, ableism casts non-normative ways of being and doing in relation to time as problem and the disabled bodymind as incapable or a burden in spaces dominated by normative orientations to time.

Developmentalist and neoliberal discourses have had a particularly strong influence on dominant understandings of time, and living well, in Euro-Western cultures. Of relevance for disabled people are understandings of time in a more macro sense in relation to life trajectories and in a more micro sense in relation to everyday experiences and uses of time. Euro-Western developmentalist orientations to human development and normative life trajectories intersect with ableism in how they valorize achieving particular milestones—graduating high school, pursuing post-secondary education or training, and paid employment to contribute to society—at particular points along a linear trajectory of time. Several studies with young people with complex care needs have reflected normative developmental and temporal expectations (Cook et al., 2013; Noyes et al., 2018); young people may also experience what Noyes et al. (2018) called “conflicting realities” (p. 2873), such as a desire to live in the moment when they were expected to plan for their future or to become independent in the context of dependence (Gibson et al., 2009; Joly, 2015; Noyes et al., 2018).

Numerous scholars in critical rehabilitation and disability studies fields have critically interrogated developmentalist assumptions underpinning structures and practices in health and social care (Fadyl et al., 2020; Gibson et al., 2014; Hamdani et al., 2015; Straus and Brown, 2021). They point to the potential and actual harms of an uncritical uptake of developmentalist discourses (e.g. in relation to what should be done as an adult) in supporting disabled young adults to live well over time when their experiences, choices, and realities may not align with normative developmental standards. The effects of developmentalism and time in relation to how young adults living with HMV compose their lives, however, has received little attention (Gibson et al., 2009, 2014).

Neoliberal discourses, as they operate in conjunction with ableist discourses, privilege time as a quantifiable and standardized resource that ideologically upholds *normative* bodies and experiences and productivity at all costs for contribution to society (Abrams et al., 2020; Kafer, 2013; Katzman et al., 2020). These discourses define good uses of time as those that contribute toward movement along normative developmental trajectories, achieving financial independence, and contributing to society, with an emphasis on productivity and efficiency. In these discourses, disability is often cast as a lack of production-oriented capacity, perpetuating a devaluing activities such as caring for the body or leisure (Shildrick, 2020). When time and its uses are understood in this way, the experiences those whose bodies differ from what is considered *normal* and the variations on temporalities of living well in everyday life are often overlooked (Kafer, 2013; Katzman et al., 2020).

Over the last decade, critical disability scholars have sought to reimagine these dominant orientations to time through the concept of *crip time*, which problematizes normative narratives of time that assume development toward independent and productive adulthoods and expectations of what can and should be done in time that are based on Euro-Western constructions of *normative* bodyminds (Kafer, 2013; Katzman et al., 2020). Crip time suggests a reorientation to time that attends to natural variations in bodyminds and what is needed to accomplish activities and goals (Katzman et al., 2020). Operating on crip time is not only about accounting for slower paces, but also about recognizing and anticipating variations in how people live in time (Kafer, 2013). While the concept of *crip time* has gained significant momentum in disability studies, health research, and especially studies with young adults living with HMV, has rarely explored experiences of time (Abrams et al., 2020; Gibson et al., 2009).

Overall, while research focused on the experiences of young adults living with HMV is emerging, living well has received little attention in the literature despite the centrality of this concept in HMV research and practice discourses. The importance of critically examining the influence of assumptions and discourses about time and its uses that contribute to dominant understandings of living well and what people can and should do as adults highlights a further need for research that broadens understandings of time in disabled young adults’ experiences.

## Methodology and methods

The paper focuses on findings derived from a larger critical narrative study investigating the experiences of young adults living with HMV. Critical narrative inquiry brings a critical theoretical orientation to narrative inquiry methodology (Clandinin, 2013; Clandinin et al., 2016). The research aim was to generate knowledge about how young adults’ experiences of living with HMV are shaped by relationships, social conditions (e.g. built environments and financial, material and human resources), discourses, and other social structures (e.g. local, provincial, national and international institutions and policies, cultural norms, and social values). The larger study was theoretically informed by writings on how *power, discourse, and ideology intersect* to shape the social conditions and structures with which people live (Fairclough, 2010; Purvis and Hunt, 1993). When referring to discourse in the study, we referenced how meanings are constructed in language and images and how they act to produce the social structures and conditions within which we live. We focused on noticing in the data how discourses are enacted through everyday living; for example, in how one narrates their experiences, how identities are discursively and materially constituted, and how space is organized or policies are constructed (Fairclough, 2010). Building from understanding how discourse functions, we then oriented to ideology, specifically for how discourses power relations operate to reinforce unequal power relations (Purvis and Hunt, 1993). We focused our analysis on deciphering how, within the participants’ narratives, dominant talk and text may be exerting *ideological effects*, places where power can be seen to be operating to promote or erase specific understandings or conceptions of living well with HMV.

This critical narrative approach provided the ground for exploring participants’ stories and experiences, while also accounting for how social contexts were woven into their lived and told stories. The inductive data analysis was deepened through our focus on inquiring further into the social conditions and structures that become evident when centering how discourse, power and ideology contribute to the dominant social order or ways to creatively resist it (Hardin, 2001; Jørgensen and Phillips, 2002; Purvis and Hunt, 1993; Souto-Manning, 2014).

### Recruitment and sampling

The study was approved by the University of British Columbia Behavioral Research Ethics Board. Young adults were eligible for the study if they: (1) were 16–40 years old; (2) used a ventilator on a daily and/or nightly basis (via tracheostomy, mask, or mouthpiece); (3) were able to communicate their needs, wishes, and experiences verbally or through augmentative or alternative communication (AAC) modes. Young adults were excluded if they: (1) were unable to understand English or (2) used Continuous Positive Airway Pressure (CPAP) ventilation via mask at night only. Participant recruitment involved convenience and snowball sampling approaches through home ventilation programs in one Western Canadian province and a home care agency in one Western U.S. state. Two broad geographic regions were chosen as it was anticipated that this approach could generate a larger sample size. Overall, five young adults (ages 18–40 years) participated in the study, four of whom identified as men and one as a woman (Table 1). Four identified as white and one as of mixed ancestry. All participants communicated verbally without the use of AAC.

**Table 1.** Participants.

Participant	Summary^a^
Andrew	Andrew was in his late-30s at the time of the study, began using a ventilator via tracheostomy in his mid-teenage years, and had experienced physical impairments, which changed over time, for most of his life and used a power wheelchair. He lived in his own single-family home. Andrew shared stories of relationships with carers and friends, especially when he first acquired the ventilator. His stories emphasized the many ways in which he was being creative(ly) in everyday life and in relation with technology and others. These stories further highlighted the many ways he found to spend time outside his home and try new things.
Jessica	Jessica was a university student in her late teens living in residence who experienced a condition that impacted her neurological drive to breathe when she slept. She did not report experiencing any physical impairments. She used non-invasive BiPAP ventilation at night, but could go without the ventilator some nights if needed. Her narratives primarily focused on her life experiences aside from ventilator use, in which she was being and becoming as a teenager and young adult, only discussing the ventilator when asked.
Jeffrey	Jeffrey was in his early 20s at the time of the study, began requiring the ventilator via tracheostomy as a child, and experienced physical impairments that changed over time and used a power wheelchair. His narratives emphasized the importance of family. He also shared stories of his experiences with community programs and other activities, some of which he framed as the “right” kind of activities while others were focused on leisure activities of meaning to him. He also shared challenges he experienced with inaccessibility and how he often needed to be creative to participate in activities outside his home.
Eric	Eric was in his mid-20s at the time of the study, experienced quadriplegic paralysis and used a ventilator via a tracheostomy and a power wheelchair. He lived in his own apartment. His stories reflected narratives of transformation and reinvention after his paralysis, especially in relation to his engagement with sports and friends. Through stories about the photographs he took, he made visible the complexities of being in relation with multiple technologies for moving and doing in the world and integrating care of the body into his routines.
Liam	Liam experienced a spinal cord injury in his late twenties and had been using a ventilator via tracheostomy for over 10 years, as well as a power wheelchair. He had lived primarily in a residential care setting since his injury. Liam shared stories of the transformations after his injury. He continued to focus his time and energy on keeping his body as healthy as possible. His narratives made visible how the institutional structures influenced his choices and opportunities with regards to caring for his body/mind. His stories of pursuing a career and improving his financial situation also highlighted social contextual influences on his opportunities to do so.

BiPAP: bilevel positive airway pressure.

a Names are pseudonyms. Some specific details are not provided (e.g. exact ages) in order to protect anonymity while still honoring participants’ stories.

### Data Generation

Data generation occurred virtually using Zoom (Zoom Technologies Inc., 2020) during the first year of the COVID-19 pandemic. Co-constructing rich and detailed narratives with participants involved ongoing negotiations of relationships and methods, which were facilitated through engagement across multiple time points during autumn of 2020 through spring of 2021. Data generation began with the telling of stories through an initial conversation and interview to develop rapport and begin to learn about participants’ experiences.

The second phase of data generation involved photo-elicitation, which offered an opportunity for participants to share stories that extended beyond the specific temporal and spatial context of an interview (Teachman and Gibson, 2018; Woodgate et al., 2017) and for broadening and deepening analysis of young adults’ experiences as they were embodied and embedded within social contexts. At the end of the initial interview, the photo-elicitation procedure was discussed; written instructions were also provided. The participants were asked to generate a collection of photographs that reflected their everyday lives, what was meaningful or distressing in their lives, and what made their lives easier or harder. In bringing together their collection of photographs, the participants took their own photographs and/or drew on existing photographs from personal or family collections. Most participants took approximately 3 weeks to generate the collection of photographs; one took 3 months.

The photographs were then reviewed by the first author and flexible guides for using the photographs to elicit the sharing of stories were developed and tailored for each participant. The guides included adaptations of prompts commonly used in such interviews, including “what was your intention when you included this photograph?,” “what is going on here?,” and “what is not depicted in the photographs?” (Teachman and Gibson, 2018; Woodgate et al., 2017). Follow-up questions from the previous interview were also integrated to further enrich the data. All interviews were audio- and video-recorded and transcribed verbatim.

### Analysis

A critical narrative analysis was undertaken attending to each participant’s stories separately and then constructing a discursive analysis looking across participants’ stories, a more detailed account of which is offered elsewhere (Straus et al., 2023). This analysis was informed by Clandinin’s (2013) approach to analysis in narrative inquiry, emphasizing temporality, sociality, and place, and integrating the critical orientation to discourses, ideology, and power discussed earlier. The analysis of discourses began by identifying particular discourses in participants’ stories through attention to language use in their storytelling and how discourses were operating in the situations they recounted (Purvis and Hunt, 1993). Annotations were made in the transcripts, attending to discourses identified and their ideological effects, where and how dominant discourses were being reproduced or resisted, and the connection to social structures and conditions. Narrative accounts—summaries of analysis of individual narratives (Clandinin, 2013)—alongside analytic notes and mind maps were used to document interpretations of these complex influences.

As the analysis evolved, we noticed the ways in which attention to temporalities intersected with certain discourses that surfaced across participants’ narratives. This led us to consider how developmentalist and neoliberal discourses of time were entangled with ableist discourses, their ideological effects, and how participants were resisting these discourses.

Reflexivity was central to constructing quality throughout data generation and analysis. Taking a reflexive stance involved consistently (re)inquiring into who one is and is becoming in researcher-participant relationships in context and into power relations in research relationships and interpretations (Clandinin, 2013), as positionalities and relations are always becoming. This ongoing process of (re)inquiring was documented through extensive reflexive notes, which contributed to an audit trail of data generation and analysis processes. All authors were licensed in the health professions (nursing, occupational therapy). The first author, who conducted interviews and led the analysis, experienced shifting embodied and enminded relations with disability throughout the study; at the time of the writing of this paper, they identified as disabled and neurodivergent.

## Findings

The analysis made visible how participants were negotiating and resisting developmentalist, neoliberal, and ableist discourses of time in terms of societal expectations on what people should do as adults and when, and in terms of expectations of uses of clock time each day. The ideological effects of these discourses were material and social and were often felt by participants as constraining. Participants also resisted these discourses and found creative ways to live in/with time differently and pursue activities that were meaningful to them.

### Negotiating normative expectations: Ableist, developmentalist, and neoliberal temporalities

Developmentalist and neoliberal discourses of time were evident in how participants engaged in decision-making in relation to what are understood as *developmentally-appropriate*, *good*, and *productive* uses of time in North American societies. Most participants who used a ventilator via tracheostomy articulated activities and aspirations that in some way reproduced these discourses in their pursuits of meaningful and paid work to contribute to society, recognizing that financial security would confer advantages in their lives. Jessica, who only used the ventilator through a face mask interface at night, did not identify as disabled, and was a student at the time of the study with a goal of pursuing a career. Her stories illuminated ways that she reproduced these discourses; time and opportunities for work appeared to be more taken-for-granted in her stories as she was able to fit in with ableist and developmentalist norms.

Jeffrey did not pursue paid work or other activities that would be considered contributions to society in neoliberal terms, but did often navigate the tensions between what was the “right” thing to do as a young adult and what he wanted to do.

Nowadays, I’m in the community transition program. [. . .] They’re supposed to prepare you for adulthood and what it’s like being an adult, teaching you — the *right* kind of experiences – teaching you to manage your money, teaching you about healthy relationships, all that fun stuff (Jeffrey, emphasis added).

While Jeffery indicated they enjoyed the social opportunities that this program afforded, the positioning of the program activities as the *right* kind of experiences conflicted with his desire to pursue activities that are termed to be “leisure.” This conflict led Jeffrey to feel isolated and uncertain about his decisions concerning use of time. For participants who did pursue paid work, the ideological effects of developmentalist and neoliberal discourses were also materialized in structures and practices that constrained their access to opportunities to pursue “work”-related goals.

#### Negotiating living with/in time as resource or commodity

Tensions between participants’ experiences and neoliberal (and ableist) societal expectations of uses of time surfaced in participants’ stories. Participants experienced a stretching and compressing of time as they negotiated what they could do in a neoliberal world in which time becomes understood as a resource or commodity. Participants felt time as compressed given that many activities, including personal care, took significantly longer than what is considered *normal.* This meant they had limited time available to do other activities such as those that were income-generating or gave them purpose. Yet, caring for the body was essential for having the energy to engage in other activities.

I don’t usually eat until later, kind of like an early lunch or around lunch is when I first start eating [. . .] between then on, it’s personal care, grooming, bowel care, ventilator and trach care, and [. . .] depending on the day, I don’t usually start anything until afternoon because I do like to plan my day out [. . .] emails, appointments, banking, you know, got to pay my employees and do paperwork and all that kind of crap. And then [. . .] personal care or physio, stretching, range of motion, I don’t usually do that until early evening (Eric).

Eric’s time was also structured in ways that attended to his body as he experienced nausea at times throughout the day that influenced opportunities to eat. Not only did activities like moving and caring for the bodymind take longer, but they also required time for anticipatory planning. This meant planning when to do different activities, anticipating how long each step would take and in which order each activity should be done in order to minimize fatigue and time spent, taking into account complex relations between bodymind, others, contexts over time. With this compressing of time and availability of time to engage in work and other activities, participants often found themselves stretched in terms of what they could feasibly do. In this way, tensions surfaced between participants’ experiences and neoliberal and ableist social structures and expectations concerning work and time that assume *normative* bodies in how they move and do in the world in time.

Tensions related to the value of certain uses of time further surfaced in participants’ stories in relation to the structures influencing management of carers. Several participants utilized directly-funded, or self-managed, home care services, in which the disabled person is provided with funding to pay their own staff to support them at home and in the community. While such services are meant to increase autonomy and choice for the individual (Kelly et al., 2021), they also reproduce neoliberal logics that offload responsibility to the disabled person to manage, hire, and pay their staff, which required a significant time commitment with no monetary compensation for themselves. This work required to manage carers, which Eric described as akin to “essentially running a small business,” not only consumed clock time, but also devalued participants’ time and energy commitments. Simultaneously, they were also negotiating a societal expectation to work and a desire for a higher income, while unpaid time spent managing carers contributed to constraints on what they could do while often living on limited disability income assistance.

The challenges participants encountered with improving their financial situations were directly connected to neoliberal assumptions about financial need for disabled persons—that is, that disabled adults should not want or need any finances beyond that which covers their basic needs. Liam highlighted the limitations imposed by policies for disability income assistance and costs for residential care when he tried to start a small business with a friend—if he paid himself through that business, he would lose access to other services, with a substantial portion of his remaining income going to residential care. This left him with little money for other activities and a desire but little incentive to work, alongside feeling compressed in terms of time for paid work.

#### Living and working in/with time in an ableist world

**Figure 1.**
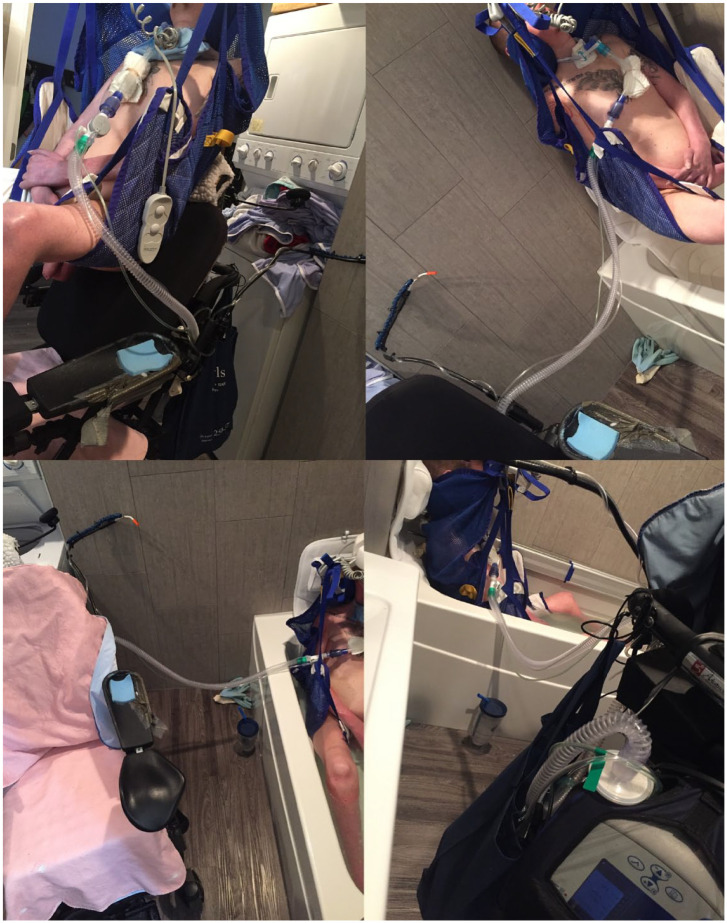
Series of photographs by Eric. ALT text: A collage of four photos of Eric transferring into the bathtub. Top left: a man with a ventilator in a Hoyer lift sling in a tight space, with a washing machine immediately behind him; Top right: man with a ventilator in a Hoyer lift sling hovering about a foot above the bathtub, with ventilator tubing stretching to the left toward a wheelchair. Bottom left: power wheelchair with pink towel on the left and the edge of a body with a Hoyer lift underneath in the bathtub, but ventilator tubing going between them; Bottom right: a man partially submerged in a bathtub with a Hoyer lift sling holding him up and ventilator tubing running from him to the ventilator on the back of a wheelchair, which is very close to the sink and bathroom leaving little space to maneuver.

Ableist discourses as they materialized in built environments and social spaces were of particular significance as participants negotiated time in living and working. Negotiating these ableist built environments contributed significantly to experiences of time to complete tasks and time available for other activities of interest and value. For example, Eric used his collection of photographs to story through everyday movements, such as the process of getting into the bathtub (Figure 1) in ways that attune us to how the amount of time spent is not uniquely a product of the impaired or disabled body, but a result of constraints produced as the impaired or disabled body comes into relation with (ableist) socio-material worlds. Indeed, bathing was a task for Eric that consumed a significant amount of time, in part, because of the ways in which he had to navigate the tight spaces of built environments designed for normative bodies. The effects of ableist environments are seen here in how, since Eric was unable to change the built environment, he would take full baths much less frequently.

**Figure 2.**
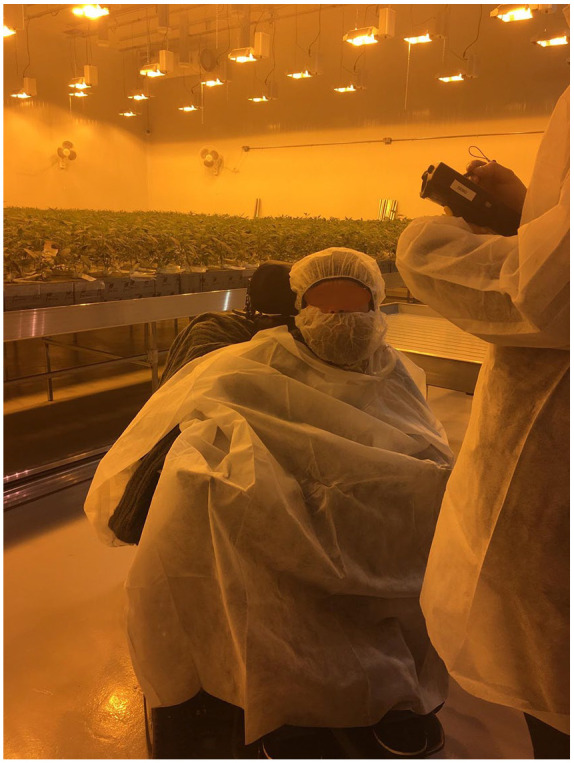
“Last Job” by Andrew. ALT text: A man in a large power wheelchair with disposable gowns draped awkwardly over his body and wheelchair, with another person with a disposable gown in the right foreground. The room is dimly lit by yellow/orange light and plants are visible in the background.

Ableist discourses as they materialized in policies and built environments further supported or constrained several participants’ time and opportunities to pursue work. Andrew spoke of encountering spaces and policies that did not anticipate a disabled body with a large power wheelchair and ventilator. For example, in his last job he encountered policies requiring him to wear personal protective equipment (PPE) when visiting the job site (Figure 2).

I was the IT manager for a medical marijuana production facility. . . . It was hilarious because of all of [the] rules and regulations on cleanliness. So everything has to be gowned. So I literally had one gown in the front, one in the back, one on the side, like trying to create this bubble boy, and yeah, it was ridiculous (Andrew).

As with built environments, policies and the PPE itself contributed to the time and effort needed to access the spaces required in his work, even with Andrew’s creative solutions.

Ableist discourses materializing in structures and attitudes further constrained Eric’s opportunities to use his time to engage in his desired work with sports organizations—with which he used to participate as an athlete prior to becoming paralyzed and now contributes to the teams through coaching, personal training support, etc.—that he hoped would ultimately lead to more paid work opportunities.

Some sports that I participate in are a little easier because they’re either outside or if we’re in the gym, there’s enough open room where I can find a place or I can get to an elevator or something. But for the most part, it is pretty tough for me to get into one of those places when their focus is on able-bodied athletes (Eric).

Eric’s stories illustrate how the spaces where he wanted to work did not anticipate disabled bodies entering those spaces. Eric also encountered ableist structures and attitudes in the selection of a location for meetings that did not have an elevator. In this case, ableist built environments intersected with ableist attitudes, which surfaced in the decision of where the meetings would be held and were not necessarily noticed by others, to constrain opportunities when Eric wanted to dedicate time to certain activities. In this way, ableist built environments and social attitudes often complicated or constrained how participants wanted to or could use their time.

### (Re)Creating temporalities of living and “working”

Participants also resisted ableist, neoliberal, and developmentalist discourses and expectations for uses of time in creative ways. As mentioned earlier, recognizing that financial security conferred advantages and with desires to have purpose in their lives, participants creatively negotiated time in ways that valued caring for the body and other health care (e.g. appointments, advocacy, management of carers and equipment) and leisure activities (e.g. connecting with friends, reading, watching movies, etc.), while creating opportunities for work or other income-generating activities that fit within their lived temporalities. Andrew started his own consulting company focusing on disability and access in travel and worked with a disability technology outreach organization that enabled the flexibility in time he needed. Liam, whose opportunities were constrained by disability income and residential care policies, invested in the stock market, an activity that he could do from home on his own schedule that gave him purpose and offered income potential while not impacting his financial relationships with residential care.

I started learning as much as I can about the market because I’m trying to change my financial situation. [. . .] Right now, I have a purpose. I’m getting up [at 6am] and I’m trying to make money every day in the market [until 1pm]. [. . .] I actively trade, on and off [. . .] I’ve been studying new strategies, things like that. [. . .] but a lot of it is dependent on my quality of sleep. (Liam)

While these work experiences approximated some ideals of paid work, they were also flexibly constructed, stretching and reorienting to time in ways that enabled meaningful activities while still improving their financial situations.

**Figure 3.**
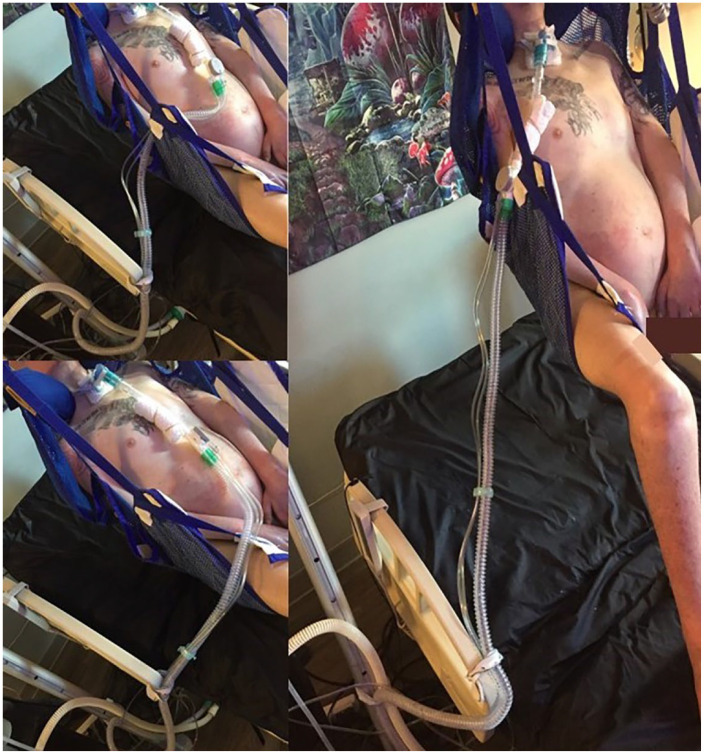
Series of photographs of transferring trial and error by Eric. ALT text: A collage of three photographs representing the trial and error of transferring, two on the left and one on the right. Top left: a man with a tracheostomy and ventilator in a hoyer lift sling elevated several inches above a bed. The ventilator tubing runs from the tracheostomy straight down to the upper abdomen and is threaded to the left through the strap of the sling. Bottom left: a man with a tracheostomy and ventilator in a hoyer lift sling elevated several inches above a bed. The ventilator tubing runs from the tracheostomy straight down to the lower abdomen and then runs to the left outside the sling straps. Right: a man with a tracheostomy and ventilator in a hoyer lift sling elevate several feet above a bed. The ventilator tubing runs directly from the tracheostomy diagonally off to the left and is stretched out fully with no further slack available.

Participants’ creativity and resourcefulness were particularly evident as they negotiated time in their everyday lives. Through trial and error, participants took time to find creative and less time-intensive ways of moving, positioning, and doing other personal care activities (Figure 3).

*Eric*: . . . most of the stuff I do is the easiest or the most simple way, and then either tied with that or coming second is comfortability. But usually, I do things the quickest and easiest way. Some things are just long and tedious, but I try to just shorten that down as much as I can by trying to be efficient or trying to be quick.

*Researcher*: Yeah, that makes sense. And if you can keep it simple and keep it efficient, then you have more time for other things?

*Eric*: Yeah, exactly.

While this approach still reflects, to some extent, neoliberal discourses of efficiency and time as commodity, participants creatively adapted practices learned from medical contexts, like transferring from bed to wheelchair to bathtub, to account for the different temporalities and relations with socio-material worlds, which became acts of stretching and compressing time.

### Imagining Futures

Developmentalist discourses of time also emphasize thinking about and planning for the future and, while most participants did imagine their futures in various ways, Jeffrey resisted imagining the future, initially expressing some frustration when asked, as if this was a question he was asked frequently and was tired of answering.

I’m going to tell you what I’m telling everyone that asks me that question. I have no idea. I have no idea. I don’t have a plan right now. I never really had a plan. . . . Not true. I had some motivation to go to college and learn something, and I lost all my motivation to do that, even before the pandemic (Jeffrey).

He instead valued spending his day playing games, watching television, and going for outings in the community, living 1 day at a time.

Other participants imagined futures full of meaningful activities, purpose, and improved financial situations. Participants who used ventilators through a tracheostomy were particularly silent on their futures in relation to life expectancy with their cyborg bodies and the technologies that were life-sustaining in a biological sense. While this could be seen as a self-protective practice, it could also be seen as an act of resistance of ableist and biomedical discourses that construct disabled bodies requiring life-sustaining technologies like ventilators as without futures, on borrowed time, or no longer able to contribute to society (Kafer, 2013). Participants’ stories of their activities and aspirations open up possibilities for understanding time and futures beyond ableist discourses.

## Discussion

The discursive analysis of participants’ narratives revealed the importance of considering living well, disability, and ventilator use relative to developmentalist and neoliberal discourses of time. In this study, the participants were becoming in and moving through time in normative and non-normative ways. Neoliberal and developmentalist discourses were analyzed to indicate how they operated to produce tensions between societal expectations regarding uses of time as an adult, such as prioritizing paid work, and their experiences and temporalities. Participants often experienced time as compressed when some activities took longer than what is considered *normal* and limited time that could be dedicated to work and contribution to society. Their narratives also revealed that what a ventilated body can do over time is not solely rooted in the limitations of the impaired and ventilated body. What a ventilated body can do is also shaped in relations with others and ableist and neoliberal socio-material worlds. A stretching of time and our understandings of it were evident in participants’ creative negotiation and reorientation of time in ways that valued activities beyond paid work while constructing opportunities for paid work or other meaningful activities in their lives. Participants also resisted ableist discourses that situated those who require ventilators as futureless, imagining futures full of possibility.

Our analysis suggests that young adults living with HMV may experience effects of—while also resisting—discourses of time at multiple, but entangled “temporal registers” (Abrams et al., 2020: 55). Most often in studies with young adults living with progressive conditions such as Duchenne muscular dystrophy and other neuromuscular conditions, temporalities are explored in relation to uncertain futures embedded in the logics of developmentalism in which the temporary nature of paid work or other activities was emphasized (Abbott and Carpenter, 2014; Abrams et al., 2020; Gibson et al., 2009; Hamdani et al., 2015). In our study, the logics of developmentalism and neoliberalism were operating in ways that situated normal life trajectories as involving and prioritizing paid work. For the most part, participants, like in Gibson et al.’s (2014) study, left this notion of pursuing paid work as unquestioned, with the exception of Jeffrey, whose stories of resistance focused on living in the moment and pursuing meaningful leisure activities. Decisions to pursue paid work were also connected to neoliberal and capitalist realities that income beyond limited disability income assistance confers advantages. These findings did not surface in previous studies with young adults living with neuromuscular conditions and/or HMV (Abbott and Carpenter, 2014; Fadyl et al., 2020; Gibson et al., 2014; Hamdani et al., 2015).

Disability communities have long critiqued structures for disability income assistance, the effects of which are essentially legislated poverty (Smith-Carrier et al., 2017). The structures governing these supports are heavily imbued with ableist and neoliberal discourses that position disabled people as not worthy of financial support if they are not contributing to society. Further complicating the financial situations of disabled persons like those who participated in this study are the often-substantial extra costs incurred, such as out-of-pocket healthcare expenses and transportation costs (Goggin and Ellis, 2020; Mitra et al., 2017), that are disproportionately higher than costs incurred for similar basic needs by non-disabled persons. Given the disconnect between disability income supports and the extra costs of living with a ventilator, limited options to promote financial security, and a desire to be seen as valued citizens, it is unsurprising that most participants wanted to use their time to pursue paid work or other activities that generated income. In this way, the study findings contribute to understandings of the potential influences on young adults’ opportunities to choose other ways to live in time.

Most prominent in our study were the entanglements of expectations for normative life trajectories and experiences of time in everyday life. Temporalities of living in an everyday sense—taking longer, valuing time for caring for the bodymind or other health care or personal activities—were experienced in some ways as a compressing and stretching of time. Reading these experiences of time through the lens of crip time oriented us to how the notion of “not having enough” time or something as “taking longer” is relational in the sense that we can only understand what is enough or what takes longer through an understanding of normative expectations of what should be done in time. In this way, our analysis rendered visible how ableist and neoliberal discourses shape how we understand time. What a ventilated body can do in any particular timeframe, whether reproducing or resisting normative expectations for what can and should be done in time, is not simply a result of the impaired body. Instead, it is produced through relations of bodies, others, and ableist and neoliberal socio-material worlds.

Our analysis also surfaced the often-invisible work involved with hiring and paying carers. Just as Katzman and Kinsella (2018) found in their study of self-managing attendant care, participants in our study described this work as similar to running a small business. While hiring and paying their own carers supported autonomy, this came with a cost of time and energy. In this study, direct funding policies seemed to render invisible the (unpaid) work and time these participants spent managing their carers, which limited time to pursue paid work or other activities that mattered to them. There are also more far-reaching risks in such positionings of work and time. As Katzman and Kinsella suggested, “requiring self-managers to contribute unpaid and largely unrecognized labor that would otherwise be the responsibility of organizations and agencies risks replicating an historical trend to require disabled people to contribute labor in exchange for access to social supports” (p. 1453). Nevertheless, making visible this often-invisible work in the context of competing demands for time and energy in young adults’ lives has potential to advance opportunities for advocacy and transformation in narratives of disabled work and valued uses of time.

The balancing of care of the body and other activities in participants’ stories points to an orientation to time wherein activities required significant planning, anticipating how long each step would take and in which order each activity should be done in order to minimize fatigue and time spent. Israelsson-Skogsberg et al. (2018) referred to these experiences as a balancing act, while Cook et al. (2013) framed these experiences of activities outside the home as a “Herculean effort” (p. 5). While the findings of this study are in some ways consistent with previous studies, reading through the lens of crip time supports a more expansive and nuanced understanding of time and planning. The process of anticipatory planning and scheduling, in which young adults were “working with and in one’s own mind/body” (Kafer, 2013: 39), required a reconfiguration of time that brought together past, present, and future to carefully plan their days. They considered their past experiences and knowledge of their body and looked into the future to anticipate what is to come to make the best decisions for them in daily life schedules. While it could be argued that all people do this to some extent, it is not something that usually takes significant time, nor is it necessarily even perceptible to most. This would mean asking critical questions about time, such as, as Kafer (2013) suggests, “how might we begin to read these practices of self-care not as preserving one’s body for productive work but as refusing such regimes in order to make room for pleasure?” (p. 39). A reorientation to time can (re)value care of the body/mind in ways that extend beyond its usefulness for promoting engagement in work that contributes to society. What if we also considered other ways of constructing time, work, and productivity toward different ends that acknowledge non-normative bodies and what constitutes productive work in ways that could enhance young adults’ choices and opportunities to live well?

Our analysis surfaced important insights concerning futures and futurities. Most participants were eager to look to the future, imagining futures full of possibility in the unspoken context of uncertain prognoses. This silence with respect to potential for decline and death in participants’ stories has been reflected in other studies with young adults living with progressive neuromuscular conditions; however, others have suggested that rarely are these young adults asked the big and difficult questions about shortened lives (Abrams et al., 2020; Gibson et al., 2009). While not every participant in this study was living with a progressive condition, using such a life-sustaining technology is often portrayed in ableist and biomedical terms as to be without futures, on borrowed time, or no longer able to contribute to society. So often, those living with progressive conditions or life-sustaining technologies struggle to imagine futures in which they can live well.

While ableist, developmentalist, and neoliberal discourses of time were analyzed to explore how dominant ideas constrain choices and opportunities for pursuing work or other activities of value to participants, participants also resisted these narrow understandings of time and found creative ways to live in/with time. In this way, their stories make visible and open up possibilities for imagining futures, understanding what a ventilated body can do in time, and the notion of time itself, in more affirming and non-normative ways. As Abrams et al. (2020) suggested, we can “make futures accessible by making them visible” (p. 56).

### Implications for healthcare practices and policy

The findings of this study highlight the importance of remaining attentive to the limits of normative discourses of time in the everyday lives and life trajectories of young adults living with HMV. Healthcare providers, especially in home and community settings, can collaborate with young adults living with HMV with whom they work to develop schedules and ways of doing together that are open to different uses of time. In addition, professionals can also integrate non-normative orientations to reframe the time required for caring for the bodymind away from hours or minutes to time well-spent and support young adults to construct their daily schedules in ways that attend to what is important to them.

The findings highlighting the effects of constructions of (non)normative life trajectories also suggest implications for programs and practices supporting young adults as they transition to adulthood and compose their lives as adults. Healthcare providers engaged in provision of transition-related services in pediatric or adult health and social care settings should consider thinking beyond the linear nature of normative adult life trajectories and integrate alternative opportunities for living well. In exploring possibilities for composing lives as adults, providers must also attend to experiences of time and explore creative ways of living in time that offer both normative and other non-normative options. Such a shift also has the potential to disrupt the ableist and neoliberal ideals of “time well spent.”

Composing adult lives with a ventilator also requires structures in place that support these aims. At the systems level, policies that address income and funding for disabled adults should be strengthened. Through integrating an equity lens that confirms the value of disabled lives and variations in uses of time, policies must acknowledge and attend to income needs and costs of living that will support opportunities for living well, such as through access to technologies, and not limit incentive to work if they want to do so. Healthcare providers can also act as allies and advocate for full participation of the disability community in the process of developing and implementing the details of any benefits in ways that address the “legislated poverty” experienced by many disabled people in the North American context.

We suggest that research with young adults living with HMV further consider understandings and experiences of time and integrate the notion of crip time and other non-normative orientations to time in their analyses. In particular, it is vital to promote increased understandings of the “in-between-ness” of living with life-sustaining technology, such as a ventilator, in relation to contemplation of potential physiological decline and death alongside imagining futures. In our study, participants were silent on this point; however, future research should carefully construct studies that explicitly ask these “big questions.”

## Reflections and storying forward

We end this paper with reflections rather than conclusions to maintain a commitment to the relational ontology and epistemology that situated the study—stories and lives are always becoming in time. In this way, there is no final or singular story to tell (Clandinin et al., 2016). It was evident how the stories of young adults living with HMV were shaping and shaped by ableist, developmental, and neoliberal discourses of time and how reorienting to alternative or non-normative constructions of time, or crip(ping) time, could offer additional choices and opportunities for young adults living with HMV to live well. We do not suggest a single narrative of or reorientation to time. Instead, the findings of this study contribute to dialogs about crip time and the possibilities that could arise from considering different ways of being and doing in the world in time. Researchers and healthcare providers alike can play an important role in coming alongside young adults living with HMV, and disabled young adults more broadly, to co-create practices, spaces, and systems that are open to different orientations to time.
